# Enhancing drought tolerance in horticultural plants through plant hormones: a strategic coping mechanism

**DOI:** 10.3389/fpls.2024.1502438

**Published:** 2025-01-20

**Authors:** Shanxia Huang, Songheng Jin

**Affiliations:** Jiyang College, Zhejiang A&F University, Zhuji, China

**Keywords:** drought stress, phytohormone, antioxidants, oxidative damage, photosynthesis, pigments content

## Abstract

Abiotic stresses are considered as a significant factor restricting horticultural crop productivity and quality. Drought stress is a major environmental constraint among the emerging concerns. Plants have significant susceptibility to drought stress, resulting in a marked decline in production during the last several decades. The development of effective strategies to mitigate drought stress is essential for sustainable agriculture and food security, especially considering the continuous growth of the world population. Several studies suggested that exogenous application of phytohormone to plants can improve drought stress tolerance by activating molecular and physiological defense systems. Phytohormone pretreatment is considered a potential approach for alleviating drought stress in horticultural plants. In addition, melatonin, salicylic acid, jasmonates, strigolactones, brassinosteroids, and gamma-aminobutyric acid are essential phytohormones that function as growth regulators and mitigate the effects of drought stress. These hormones frequently interact with one another to improve the survival of plants in drought-stressed environments. To sum up, this review will predominantly elucidate the role of phytohormones and related mechanisms in drought tolerance across various horticulture crop species.

## Introduction

**Figure 1:**
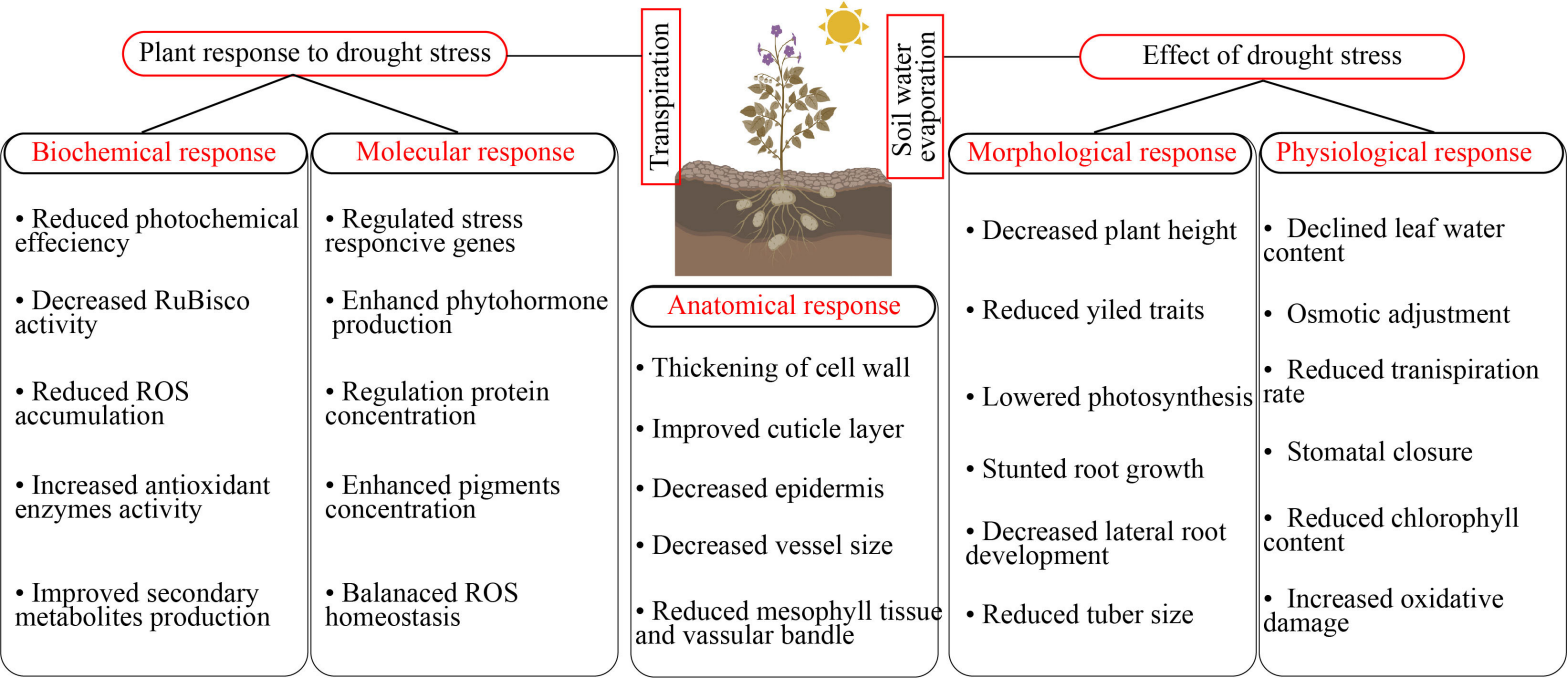
Horticultural plants responses to drought stress.

Plants are affected by abiotic stresses, prompting various internal changes inside them. These abiotic variables affect plant growth and productivity. Abiotic variables refer to the interactions between living organisms and plants that have both beneficial and detrimental consequences. Positive influences may have a favorable impact on plant development. Adverse effects considerably decreased the horticultural plant yield and productivity (Iqbal et al., 2022; Nazir et al., 2024). Plant defense mechanisms using diverse chemical components mitigate adverse effects (Zulfiqar et al., 2022). These climatic changes have intensified drought stress, hence garnering considerable attention lately. Drought is a critical factor that limits worldwide agricultural productivity (Yang et al., 2021). The intensity of the drought is escalating, leading to elevated prices for food. In addition, by 2050, the world population is projected to rise to 9.8 billion (Farooqi et al., 2020). Agricultural output must increase by 70% to satisfy the food needs of an increasing population (Cao et al., 2024). To save future generations from impending crises, it is imperative to advance technology and policies addressing climate change and drought stress, including reforestation, effective water utilization, population management, and the cultivation of drought-resistant crops (Şimşek et al., 2024). Recent years have seen substantial advancements in clarifying the molecular pathways related to drought stress responses in plants (**Table 1**; **Figure 1**).

**Table 1:** Drought stress decrease growth of horticultural plants.

Crops	Findings	References
Tomato	Lowered leaf water content, increased oxidative damage, EL level, altered antioxidant enzymes activity, and metabolites production	Altaf et al., 2022
Watermelon	Increased hydrogen peroxide (H_2_O_2_) and malonaldehyde (MDA) accumulation, damage root growth, and altered antioxidant enzymes activity	Li et al., 2019
Potato	Reduced tuber yield and quality, protected photosynthetic capacity, and enhanced chlorophyll content	Zheng et al., 2022
Watermelon	Enhanced oxidative damage by increased H_2_O_2_ and MDA concentration and reduced root growth and mineral uptake	Yan et al., 2023
Strawberry	Enhanced antioxidant enzymes activity, root growth, mineral homeostasis, and reduced oxidative damage	Safa Eynaladin et al., 2025
Apple	Altered osmotic balanced, antioxidant enzymes activity, and increased EL and MDA production	Kumari et al., 2020
Pepper	Lowered chlorophyll content, pigments concentration, antioxidant enzymes activity, increased MDA and H_2_O_2_ uptake in pepper leaf	Ardıç et al., 2023

**Figure 2:**
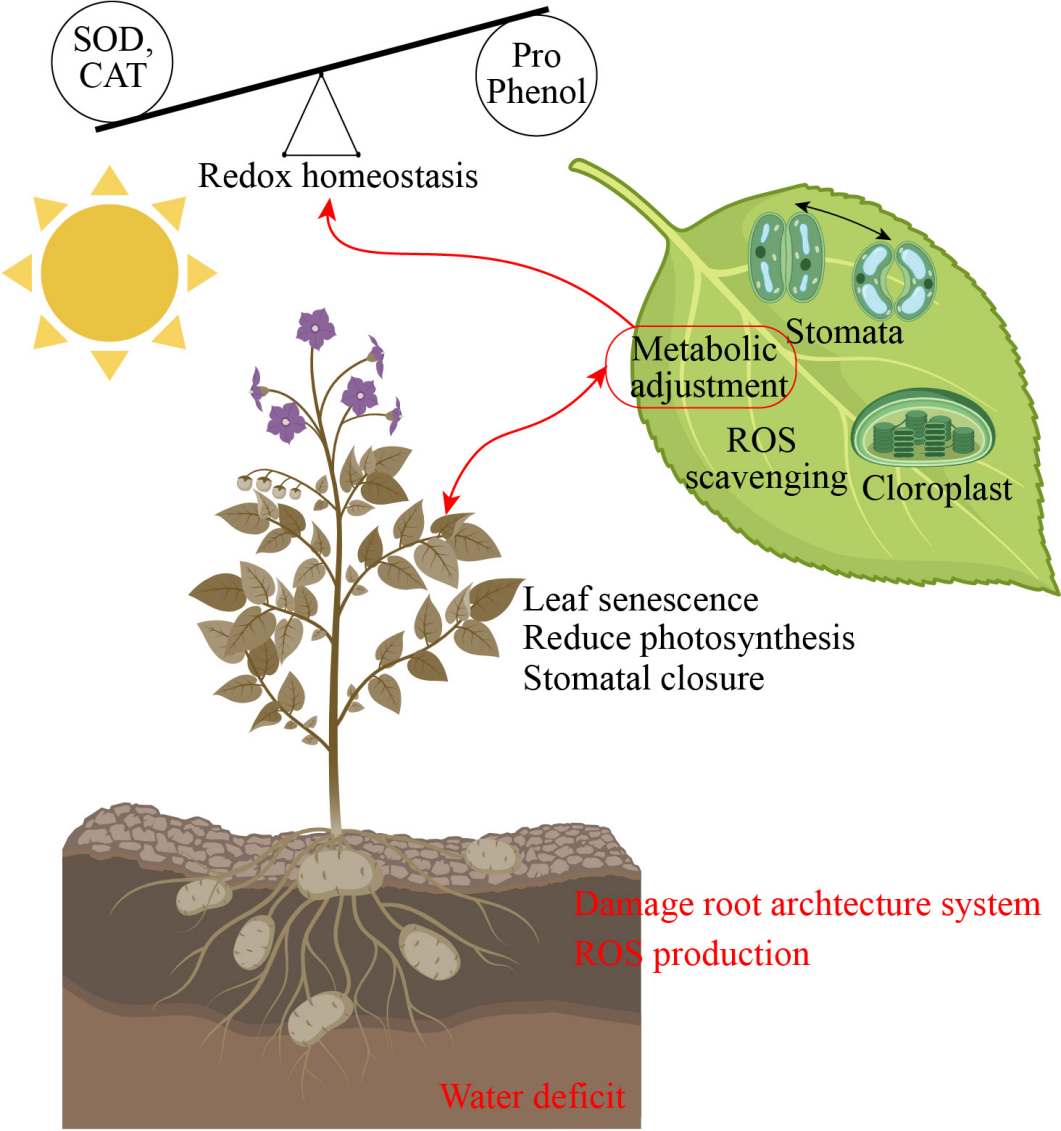
Drought stress altered redox homeostasis and leaf photosynthesis performance. SOD, superoxide dismutase; CAT, catalase; Pro, proline; ROS, reactive oxygen species.

Drought stress induces several morpho-physiological and metabolic alterations that negatively influence plant growth and yield (Sako et al., 2020). Drought stress presents a significant problem to global agriculture, requiring a deep understanding of plants’ adaptation systems. In addition to rapid physiological reactions, new research has shown the intriguing phenomena of epigenetic memory in drought-adapted plants (Kaya et al., 2024). The epidermal wax of plants inhibits non-stomatal water loss and enhances water usage efficiency, facilitating adaptation to arid conditions (Gao et al., 2024). Stomatal regulation and stress signaling are the mechanisms by which plants respond to drought stress. Plants regulate their response mechanisms through the utilization of phytohormones (Haider et al., 2024). Drought stress effectively hindered different physiological function such as leaf area, root length, stem mass, lateral root development, node number, reduced canopy size, and even cause cell death (Feng et al., 2024). Drought stress damage leaf growth, restricted leaf photosynthetic activity, reduced chlorophyll content, stomatal conductance, water potential, and reduced pigments level in leaf (Sári et al., 2024). In another study, Xie et al. (2024) reported that drought stress significantly reduced the seedling growth, pigments concentration, chlorophyll content, and antioxidant enzymes activity and caused oxidative damage in tomatoes. The leaf water potential, antioxidant enzymes activity, secondary metabolites production, proline uptake, and growth were reduced in cucumber under drought stress (Ahmad et al., 2024). Plant growth is significantly affected by water scarcity, mostly owing to the suppression of cell elongation. Water-stressed plants exhibit reduced height and diminished leaf area, resulting in less absorption of photo synthetically active radiation, a lowered rate of photosynthesis, and, ultimately, a reduced yield (Abbas et al., 2023). Water shortage induces stomatal closure, leading to limited CO_2_ uptake by the leaves and reducing the operational efficiency of Calvin cycle enzymes, particularly Rubisco, due to substrate scarcity (Nguyen et al., 2018). Due to increased photorespiration and decreased stomatal conductance, net photosynthesis is the main physiological measurement that is restricted by drought stress (Chieb and Gachomo, 2023). Excessive reactive oxygen species (ROS) production can also cause damage to the photosynthetic system when the stomata are closed, resulting in a decrease in growth and photosynthesis (**Figure 2**) (Bouremani et al., 2023).

**Figure 3:**
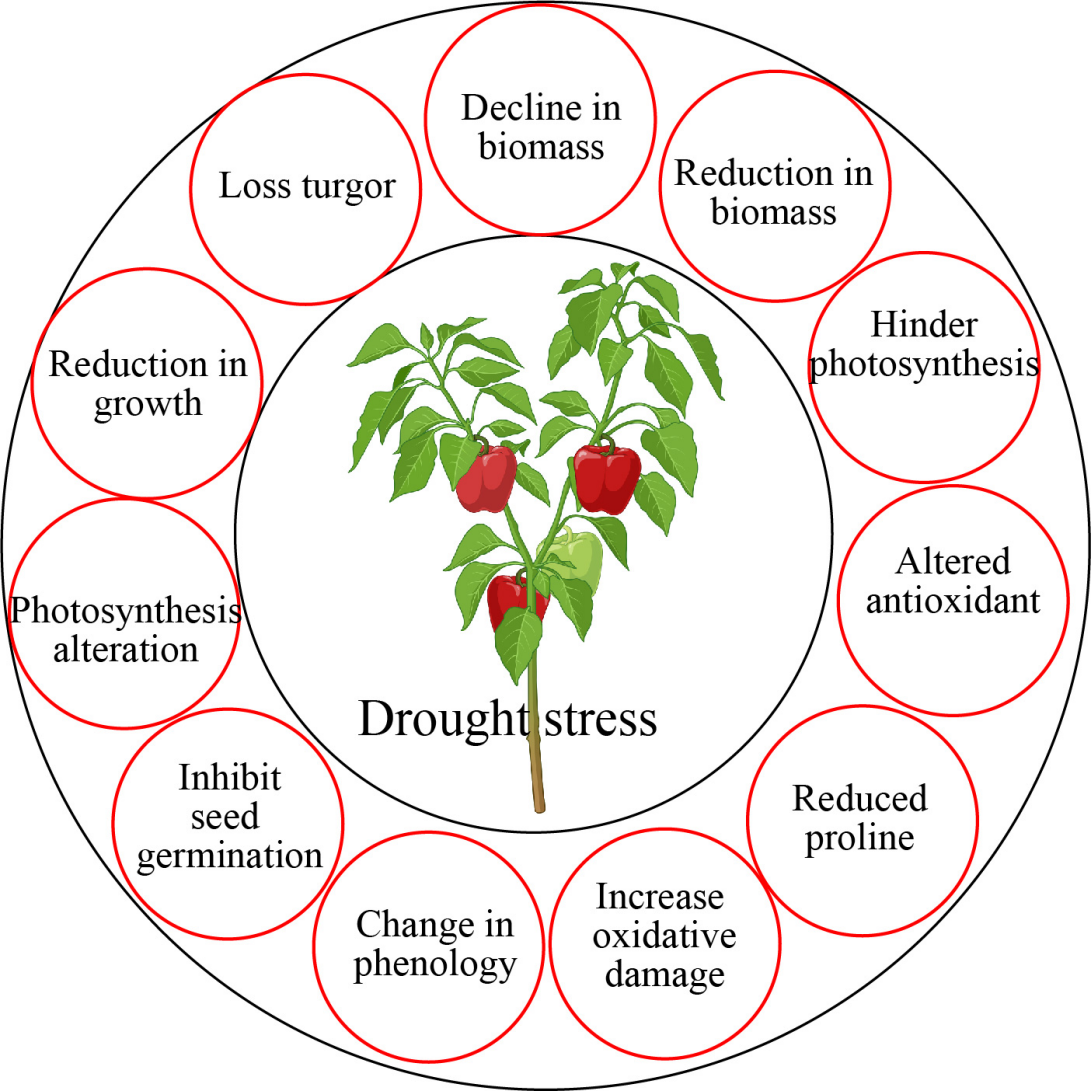
Drought stress altered wide range of physiological and morphological functions in horticultural crops.

Phytohormone is an important plant growth regulator having low molecular weight (Pandey et al., 2017). The synthesis of several plant hormones occurs in response to drought stress, regulating activities associated with drought tolerance mechanisms (**Figure 3**). Phytohormones such as melatonin, salicylic acid, jasmonates, strigolactones, brassinosteroids, GABA, auxin, gibberellin, cytokinins, ethylene, abscisic acid, glycine betaine, polyamines proline, and trehalose have a role in osmotic adjustment and enhanced drought stress tolerance mechanism (Ciura and Kruk, 2018). Drought stress also triggers the antioxidant defense system that participates in the elimination of ROS uptake in plants (Fahad et al., 2015). Phytohormones regulate wide range of functions in horticultural plants such as protected photosynthesis, lateral root development, secondary metabolites production, redox balanced, mineral nutrient accumulation, osmotic adjustment, upregulated antioxidant defense, and enhanced drought stress tolerance in horticultural plants (Ullah et al., 2018; Jogawat et al., 2021; Salvi et al., 2021).

We briefly examined the significance of phytohormones in plant growth regulation. We want to address these research questions: (i) how does phytohormone govern plant growth and development physiologically? How does phytohormone influence plant growth? (iii) Are there any ignored physiological features important for understanding phytohormone stress regulation? We addressed new scientific advances to differentiate our review from others. This work helps researchers and policymakers create efficient abiotic stress mitigation measures for horticulture crops, improving global food security.

## Potential functions of phytohormone

Endogenous plant hormones are essential for both developmental processes and the plant’s reaction to abiotic variables. The primary mediators of plant responses to drought stress are phytohormones (Asghar et al., 2022). Phytohormones are further classified according to the chemical structures of some groups (Mubarik et al., 2021). The primary stress-responsive hormone generated upon drought signal detection is abscisic acid (ABA). It is primarily synthesized in the root and then transported to the leaves to regulate stomatal opening, channel activity, and the expression pattern of ABA-responsive genes (Wani et al., 2016). Furthermore, phytohormones exist at very low amounts inside plants, complicating their measurement analysis (Checker et al., 2018). Phytohormones enhanced abiotic stress tolerance in many plant species such as tomato, radish, strawberry, eggplant, and carrot (Ciura and Kruk, 2018). Phytohormones application regulated mineral nutrient accumulation, maintained osmotic adjustment, balanced leaf water potential, reduced oxidative damage, and upregulated antioxidant enzymes activity in horticultural plants (Han et al., 2018). Several studies suggested that phytohormone application showed considerable improvement in the field of horticulture (Parmar et al., 2017).

## Phytohormones protected horticultural crops under drought stress

### Salicylic acid

Salicylic acid is a versatile plant growth regulator known to participate in plant responses to stressors (Khan et al., 2015). Foliar treatments of salicylic acid may mitigate the deleterious effects of oxidative stress induced by drought via several mechanisms (Kang et al., 2014). Salicylic acid application promotes lateral root development, increases secondary metabolites production, and boosts osmolytes accumulation, thus sustaining the water potential of horticultural plants during drought stress conditions (**Table 2**) (Ullah et al., 2018). In addition, salicylic acid may function by sustaining the overall chlorophyll concentration in plants, therefore safeguarding their photosynthetic machinery (Horváth et al., 2007). Salicylic acid alleviates the adverse effects of drought stress and functions as a signaling molecule to stimulate the gene expression pattern of stress-related genes and protein (Song et al., 2023). It was extensively documented and studied that salicylic acid directly engages in the activation of plant defense systems (Chen et al., 2023). In addition, salicylic acid has the ability to increase the production and activity of antioxidant enzymes while also activating plant defense mechanisms (Rasheed et al., 2022). Salicylic acid enhances chlorophyll content and improves photosynthetic efficiency, consequently considerably increasing crop production and other yield-related physiological indices under drought stress environment (Iqbal et al., 2022).

**Table 2:** Salicylic acid enhanced drought stress tolerance in horticultural crops.

Crops	Findings	References
Cantaloupe	Increased fruit yield and quality, antioxidant enzymes activity, photosynthetic activity, and proline uptake	Alam et al. (2022)
Cucumber	Protected photosynthetic apparatus, increased chlorophyll content, maintained photosystem functions	Baninasab (2010)
Strawberry	Increased enzymatic and non-enzymatic antioxidant enzymes activity, chlorophyll content, and net photosynthetic rate	Dakheel et al. (2022)
Sweet potato	Maintained redox homeostasis, balanced nutrient accumulation, and enhanced drought stress tolerance	Huang et al. (2022a)
Lettuce	Maintained leaf water content and osmotic adjustment and increased antioxidant enzymes activity	Kiremit et al. (2024)
Sugar beet	Protected photosynthetic apparatus, increased proline and protein content, and lowered MDA and EL in sugar beet leaves	Li X. et al. (2022)
Cucumber	Increased drought stress tolerance, lowered EL and MDA level, and improved root growth and mineral uptake	Mardani et al. (2012)

Foliar treatments of salicylic acid have been shown to enhance growth in major horticultural (tomato, potato, strawberry, and cucumber) crops under drought stress (Damalas and Koutroubas, 2021). Salicylic acid application regulates normal plant growth, such as increasing flowering, promoting bud differentiation, regulating seed dormancy, and enhancing the number of flowering (Joseph et al., 2010). Several studies suggested that salicylic acid is a multifaceted biomolecule in the response to drought stress. It can regulate the cell wall expansion, regulate hormonal production, and reduce oxidative damage in horticultural plants (Saleem et al., 2021). Salicylic acid application enhanced ion homeostasis, balanced cellular membrane integrity, and regulated antioxidant defense system in response to drought stress. In addition, salicylic acid promoting lateral root growth development, osmotic adjustment, and antioxidant enzymes activity protected photosynthesis in plants (Sharma et al., 2020). Drought stress treatment showed a considerable reduction in chlorophyll content, photosynthesis, relative water content, membrane damage, antioxidant enzymes activity, and pigments concentration in tomato seedlings. A subsequent treatment with salicylic acid mitigated water-induced stress and markedly enhanced the aforementioned metrics. Secondary metabolites production, proline content, and antioxidant enzyme concentration increased in tomato seedlings in response to both salicylic acid and drought treatments (Aires et al., 2022). Salicylic acid treatment significantly affected the growth status, net photosynthetic rate, leaf water potential, and antioxidant enzyme concentration of strawberry plants exposed to drought stresses (Dakheel et al., 2022). The antioxidant enzymes activity, osmolytes production, and growth of sugar beet increased in sugar beet plant by the application of salicylic acid under drought stress environment (Khodadadi et al., 2020). The supplementation of salicylic acid to drought-stressed seedling decreased cellular membrane damage by stimulating the antioxidant enzyme activity and maintained osmotic adjustments in two sweet potato genotypes (Huang et al., 2022a). When watermelon seedlings exposed to drought stress environment because of water deficit grow faster, as salicylic acid promotes the secondary metabolites production, increased osmolytes accumulation, and reduced electrolyte leakage level in watermelon seedling (Silva et al., 2023). Furthermore, recent molecular research has shown that salicylic acid may modulate many gene-level processes in plants, hence enhancing their tolerance to abiotic stress (Zulfiqar et al., 2022). The foliar application of salicylic acid increased growth attributes while reducing stomatal conductance under severe water shortage stress. However, significant water deficiency stress markedly increased the value of SPAD (relative chlorophyll content) index. Exogenous supplementation of salicylic acid may enhance the characteristics of cucumber seedlings and increase their resistance to water stress (Mardani et al., 2012). Salicylic acid treatment promoted yield traits, leaf water content, proline uptake flavonoids, soluble solid concentration, and balanced membrane stability in cantaloupe during drought stress environment (Alam et al., 2022). Spray treatment of salicylic acid enhanced relative chlorophyll content, prolonged fruit ripening period, and elevated secondary metabolites production and antioxidant enzymes activity in melon under drought environment (Nasrabadi et al., 2015).

**Figure 4:**
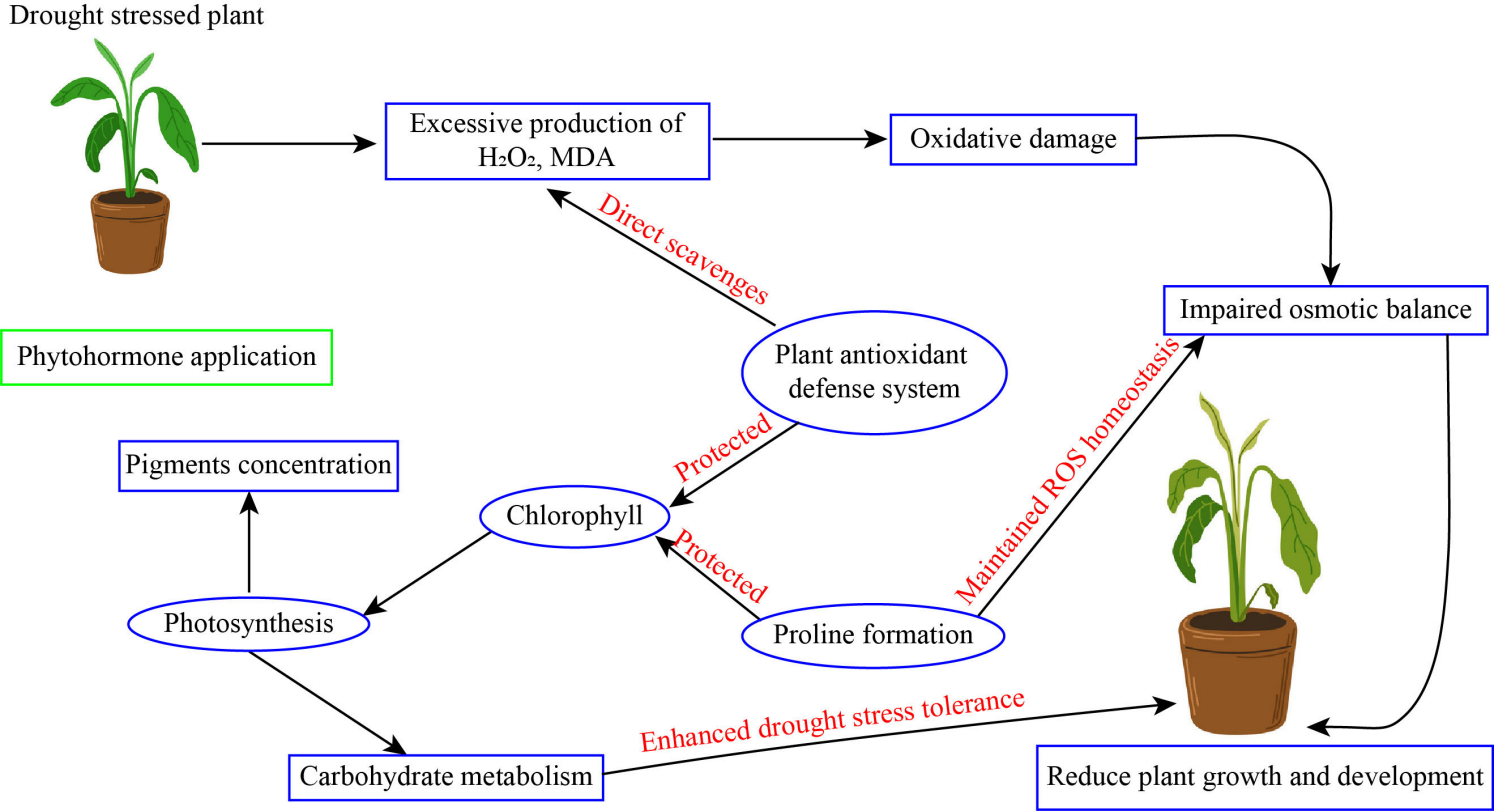
Phytohormone application enhanced drought stress tolerance in horticultural plants.

Foliar application of salicylic acid significantly enhanced growth of grape tomato seedling (Chakma et al., 2021), increased chlorophyll content in strawberry (Mozafari et al., 2018), enhanced the non-photochemical efficiency in sugar beet (Li et al., 2022), increased tuber yield and quality of sugar beet (Youssef and Abdelaal, 2023), protected photosynthetic apparatus in cucumber (Baninasab, 2010), and promoted yield and quality of watermelon (Nastari Nasrabadi et al., 2023). Continuing study into the molecular processes of salicylic acid will enhance our comprehension of plant stress tolerances. This review emphasizes the importance of salicylic acid in improving plant resistance to drought stress environment, therefore facilitating their survival and production under adverse environmental circumstances. Subsequent research in this domain may enhance the formulation of efficacious techniques for crop enhancement and stress mitigation (**Figure 4**).

### Melatonin

Melatonin is a universal biomolecule (Zhang et al., 2015). With its function in horticultural plant growth, melatonin significantly contributes to plant stress defense (Dzinyela et al., 2024) (**Table 3**). Plants often face challenges under abiotic stress conditions (Zhao et al., 2022). Numerous plant species that are abundant in melatonin have demonstrated a greater ability for maintaining stress tolerance (Debnath et al., 2019). Melatonin may assume markedly distinct functions in the regulation of plant growth and development at low and high concentrations within the same species (Huang et al., 2022b). In cherry plants, melatonin facilitates roots at low doses but suppresses growth at elevated levels in cherry tissue culture. Excessive concentrations may induce hazardous consequences (Zhang et al., 2022). This indicates that melatonin may function differently at low and high doses. Elevated levels of melatonin may significantly diminish ROS in cells, thereby influencing ROS-dependent signaling pathways and impeding cellular proliferation (Ayyaz et al., 2022). Melatonin regulates the concentrations of ROS and enhances molecular defenses that increase plant resilience to drought stress (Moustafa-Farag et al., 2020). Melatonin is a potent antioxidant compound and master growth regulator that protected plants from oxidative damage and modulate numerous responses to environmental disruptions, particularly water stress (Colombage et al., 2023). Melatonin serves as a signaling molecule at the cellular level and enhances the expression of many antioxidant enzymes, hence increasing its efficiency as an antioxidant (Raza et al., 2022).

**Table 3:** Melatonin enhanced drought stress tolerance in horticultural crops.

Crops	Findings	References
Potato	Increased leaf water potential, osmotic adjustment, osmolytes production, and antioxidant enzymes activity	El-Yazied et al. (2022)
Sugar beet	Increased proline content, tuber size, leaf area, photosynthetic assimilation rate, and chlorophyll content, and decreased H_2_O_2_ level	He et al. (2023)
Tomato	Increased mineral nutrient uptake and enhanced drought stress tolerance, leaf area, and leaf water content	Huang et al. (2023)
Cucumber	Enhanced drought stress tolerance and leaf water potential, maintained redox homeostasis, and altered antioxidant enzymes activity	Lee and Back (2019)
Kiwifruit	Increased photosynthetic efficiency and chlorophyll content, decreased oxidative damage, and enhanced drought stress tolerance	Liang et al. (2019)
Carrot	Increased leaf water potential and mineral nutritional status, balanced osmotic adjustment, and enhanced drought stress tolerance	Rosińska et al. (2023)
Pepper	Increased nitrogen accumulation, maintained ion homeostasis, regulated redox homeostasis, and reduced oxidative damage	Kaya and Shabala (2023)

Melatonin is a stress relief molecule (Tiwari et al., 2021; Ahmad et al., 2023). Melatonin application efficiently enhanced drought stress tolerance in carrot, radish, pepper, sweet potato, and cucumber (Shi et al., 2015; Raza et al., 2022). The supplementation of melatonin to tomato seedlings improves root vigor, alleviates stress-induced damage to PSII response centers, diminishes the adverse effects of dehydration by modulating the antioxidant system, and decreases the cellular concentration of harmful chemicals in the plants (Liu et al., 2015). In another study, Altaf et al. (2022) reported that drought stress considerably decreased growth, hindered photosynthetic activity, restricted pigments concentration, and caused oxidative damage in tomato seedling. In contrast, melatonin application potently increased the tomato seedling growth by recovering the above traits under drought environment. Melatonin treatment promoted lateral root development, increased seed germination, regulated antioxidant enzymes activity, and reduced oxidative damage in cucumber seedling under water stress environment (Zhang et al., 2013). Furthermore, Khan et al. (2023) reported that drought restricted strawberry seedling growth by decreasing the pigments concentration level, hindering enzymes level, and damaging oxidative stress biomarkers. In contrast, melatonin application resorted the strawberry seedling growth by decreasing oxidative damage and increasing antioxidant enzymes activity under drought environment. Foliar application of melatonin enhances the growth capacity of sugar beet plants under drought stress mostly by diminishing cellular membrane integrity level, elevating antioxidant enzyme activities, protecting photosynthetic capacity, and facilitating chlorophyll production (He et al., 2023). Ardıç et al. (2023) revealed that melatonin considerable improved chlorophyll content, antioxidant enzymes activity, and mineral nutrient content, and reduced oxidative damage by reducing the MDA accumulation in pepper under drought condition. In another study, the authors described the essential function of melatonin generation in response to osmotic stress via plant hormone signal transduction. It is shown that ABA signaling is pivotal in melatonin production under osmotic stress (Yan et al., 2023). Seed pretreatment with melatonin showed considerable improvement in the growth of carrot, seed germination, and osmotic adjustment under drought stress environment (Rosińska et al., 2023). Furthermore, Xia et al. (2020) reported that drought treatment dramatically reduced the kiwifruit growth by reducing the enzymatic activity, chlorophyll content, and pigments concentration, and increasing the oxidative damage by enhancing the MDA concentration, EL level, and H_2_O_2_ content in kiwifruit leaves. In contrast, melatonin treatment significantly recovered these traits such as protecting photosynthetic apparatus, upregulating antioxidant enzymes activity, and reducing oxidative damage in kiwifruit under drought conditions. Under drought stress environment, melatonin significantly regulated the tuber yield of potato plants by impeding ABA transfer from the root to the shoot system while simultaneously enhancing the levels of non-reducing sugars (El-Yazied et al., 2022). Melatonin remarkably promoted drought stress tolerance via regulating the leaf photosynthesis and maintained membrane stability and lateral root development in tomato (Mushtaq et al., 2022).

Melatonin application potential improved secondary metabolites production in tomato (Huang et al., 2023), regulated antioxidant defense system in cucumber (Lee and Back, 2019), maintained glyoxalase enzymes system in pepper (Kaya and Shabala, 2023), maintained osmotic adjustment in pepper (Li et al., 2019), upregulated mineral metabolism and nutritional status in strawberry (Safa Eynaladin et al., 2025), and decreased MDA and H_2_O_2_ accumulation in kiwifruit (Liang et al., 2019). Melatonin is a multifaceted biomolecule that promotes growth and yield improvement during drought conditions, making it an appropriate choice for sustainable agricultural practices aimed at ensuring food security (Zeng et al., 2022).

### Jasmonates

Jasmonates, including jasmonic acid and methyl jasmonates, are recognized for their involvement in several physiological processes (Ahmad et al., 2016). The exogenous supplementation of jasmonates evaluated on several plants under stress circumstances has shown efficiency in enhancing plant stress resistance (**Table 4**) (Ali and Baek, 2020). Jasmonates are universally present throughout the plant kingdom (Iqbal et al., 2022). Jasmonate and methyl jasmonates actively contribute to leaf senescence (Siddiqi and Husen, 2019). Jasmonates prominently increased drought stress tolerance by upregulating the antioxidant defense system and maintaining redox homeostasis, flowering, fruit ripening, and hormonal production (Per et al., 2018). In addition, physiological functions associated with jasmonic acid included seed germination, protein accumulation, leaf chlorosis, flowering development, and secondary metabolites production in horticultural plants (Santino et al., 2013; Wasternack, 2014).

**Table 4:** Jasmonates enhanced drought stress tolerance in horticultural plants.

Crops	Findings	References
Radish	Increased osmolytes production, proline uptake, antioxidant enzymes activity, and photosynthetic activity	Chen et al. (2019)
Sugar beet	Decreased oxidative damage, increased antioxidant enzymes activity, chlorophyll content, and lowered EL level	Fugate et al. (2018)
Peppermint	Increased flavonoids, phenolic content, photosynthetic activity, proline uptake, ASA, and GSH enzymes activity	Gholamreza et al. (2019)
Melon	Enhanced drought stress tolerance, balanced osmotic adjustment, lowered EL, and oxidative damage	Nafie et al. (2011)
Cowpea	Protected photosynthesis, increased enzymes activity, chlorophyll content, and enhanced drought stress tolerance	Sadeghipour (2018)
Cauliflower	Increased mineral nutrient content and leaf water content, maintained redox homeostasis, and lowered EL level	Wu et al. (2012)

The supplementation of methyl jasmonates enhances growth, promotes the accumulation of secondary metabolites, and influences endogenous hormone levels, along with other metabolic processes in stressed horticultural plants (Yu et al., 2018; Delgado et al., 2021; Rehman et al., 2023). Jasmonic acid application increased drought stress tolerance by regulating growth traits and promoting polyamines accumulation and antioxidant enzymes activity in tomato seedling under drought environment (Zhang and Huang, 2013). Methyl jasmonates application increased biomass and secondary metabolites production and stimulated antioxidant defense system in cucumber under water stress environment (Wang et al., 2022). Water stress treatment considerably decreased the pigments concentration, leaf water potential, and protein concentration, while it enhanced antioxidant enzymes activity, MDA, H_2_O_2_, and proline accumulation in strawberry. In contrast, jasmonic acid application along with drought treatment showed significant improvement in the growth of strawberry seedling (Yosefi et al., 2020). Jasmonic acid application to *Brassica rapa* significantly mitigated drought-induced damage by altering secondary metabolites production, promoting antioxidant defense system, and protecting photosynthetic machinery (Ahmad Lone et al., 2022). Methyl jasmonates stimulated the production of anthocyanin, flavonoids, and osmolytes production, regulating antioxidant enzymes activity and maintaining photosystem functions while also reducing EL level and maintaining leaf water potential, protecting leaf photosynthesis, and seedling growth. Methyl jasmonates significantly alleviated drought stress in purple basil by increasing its secondary metabolism, photosynthetic apparatus, secondary metabolism, and quality- and yield-related attributes (Lopes et al., 2024). Methyl jasmonates application positively influences the growth of radish by increasing the osmolytes production and reducing the oxidative damage under drought environment (Chen et al., 2019). Methyl jasmonates application along with water stress treatments substantially enhanced flavonoid content, total phenolic levels, and antioxidant capability in peppermint (Gholamreza et al., 2019). In another study, jasmonic acid application regulated antioxidant defense system and reduced H_2_O_2_ accumulation in *Cucumis melon* under drought environment (Nafie et al., 2011). Supplementation with jasmonic acid significantly enhanced growth status and boosted antioxidant defense system and the resilience of sugar beet under drought stress environment (Ghaffari et al., 2019). Methyl jasmonates application potentially improved leaf photosynthetic apparatus, increased antioxidant enzymes activity, and reduced MDA accumulation in cauliflower leaves (Wu et al., 2012).

**Figure 5:**
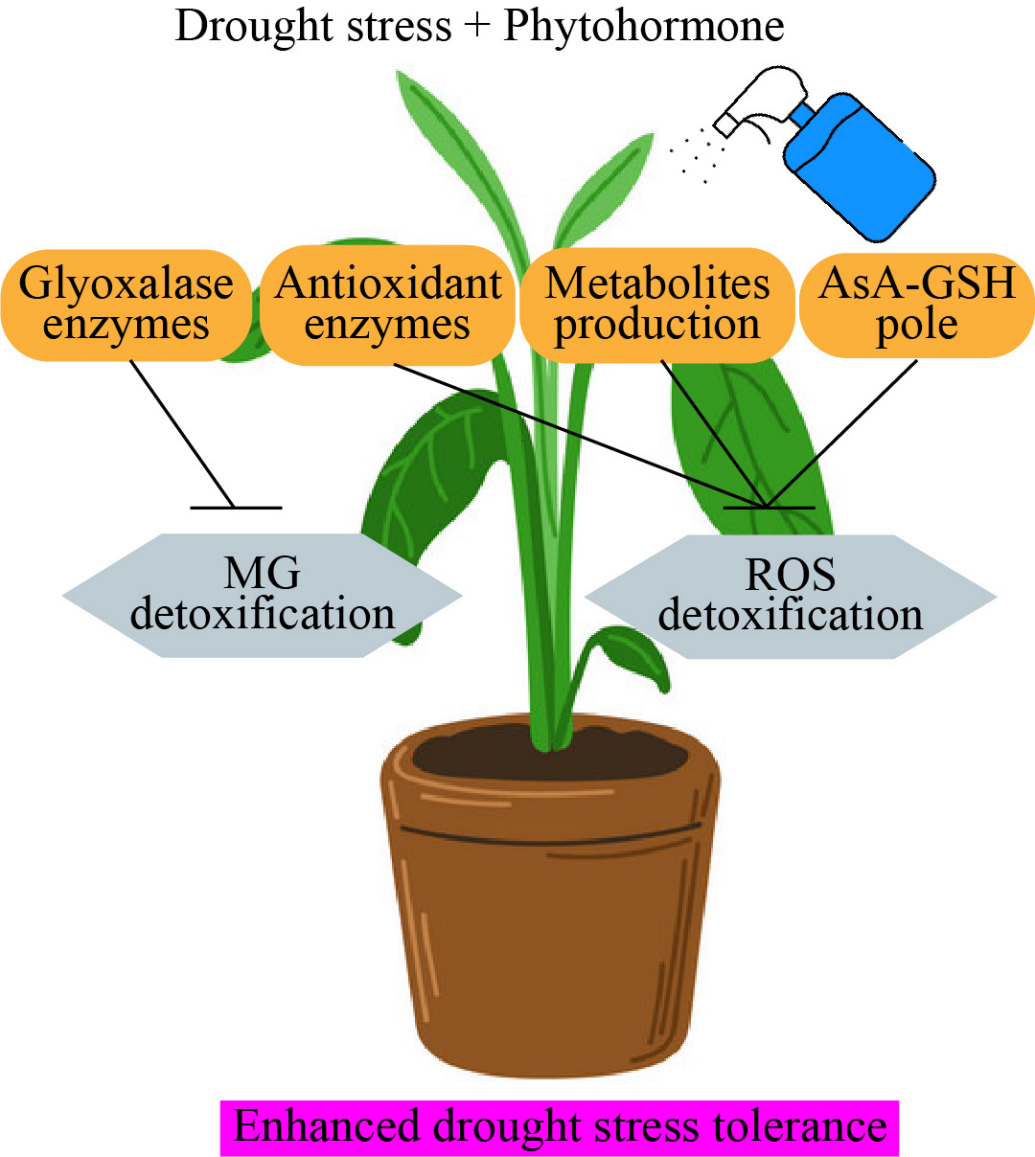
Phytohormone regulates antioxidant enzymes activity under drought stress environment. AsA-GSH, glutathione-ascorbate; ROS, reactive oxygen species; MG, methyl glycolase.

Jasmonates application promoted seedling growth of tomato (Muñoz-Espinoza et al., 2015), enhanced pigments concentration and altered leaf ultrastructure in cucumber (Wen et al., 2023), and decreased oxidative damage in strawberry leaves (Wang, 1999). Furthermore, methyl jasmonates may enhance drought stress tolerance by elevating photosynthetic assimilation rate, maintaining stomatal conductance, improving proline uptake and osmotic adjustment compounds and antioxidant activity, and reducing H_2_O_2_ and MDA accumulation in *Citrus* (Xiong et al., 2020). Fugate et al. (2018) reported that drought stress treatment dramatically decreased fresh weights and leaf gas exchange traits, damaged PSII system, declined leaf water potential, and increased oxidative damage. In contrast, methyl jasmonates supplementation significantly recovered growth status and restored the above parameters by enhancing the drought stress tolerance in sugar beet. The author described the efficiency of MeJA in boosting *Impatiens walleriana* capacity to endure water stress *in vitro*. *Impatiens walleriana* enhanced water stress resistance by stimulating defense-related metabolic processes, mostly triggered by pretreatment with the minimal methyl jasmonates concentration used (Đurić et al., 2023). Foliar application of methyl jasmonates considerably increased growth, chlorophyll content, and antioxidant enzymes activity, and decreased membrane damage in cowpea under drought environment (Sadeghipour, 2018). Seed or foliar supplementation of methyl jasmonates reinstates normal growth and morphological functions via activating the antioxidant enzymes activity under drought stress environment (Mohi-Ud-Din et al., 2021). Therefore, it may be said that jasmonates has a beneficial regulatory role in horticultural plants during adaptation to drought stress (**Figure 5**).

### Brassinosteroids

Brassinosteroids are a group of plant hormones that regulate diverse range of functions, including photosynthesis, cell elongation, flowering, root system architecture, and responses to stresses (Ahammed et al., 2015; Ali et al., 2019). BRs have demonstrated the ability to alleviate the adverse impacts of drought by regulating various metabolic functions, such as seed germination, stomatal control, leaf senescence, lateral root development, antioxidant enzymes system, redox homeostasis, osmotic adjustment, maintained leaf water potential, and nutrient absorption in horticultural plants (**Table 5**) (Bhandari and Nailwal, 2020; Zhang et al., 2023). The function of brassinosteroids in protecting plants from environmental challenges is crucial for sustained production. Furthermore, the application of brassinosteroids has dramatically decreased fruit cracking in litchi (Sharma, 2021). Brassinosteroids increased both the quantity and quality of fruit produce in several horticultural fruit crops (Ali, 2017). They also influence cotyledon development, root extension, leaf creation and growth, and plant biomass accumulation. Another key physiological reaction in plants associated with brassinosteroids activity is ethylene synthesis (Ali, 2019).

**Table 5:** Brassinosteroids enhanced drought stress tolerance in horticultural crops.

Crops	Findings	References
Radish	Decreased oxidative damage, lowered EL and MDA level, and increased secondary metabolites production	Balaraju et al. (2015)
Potato	Maintained osmotic adjustment, balanced leaf water potential, and increased proline and enzymes activity	Guo et al. (2024)
Tomato	Increased pigments concentration, photosynthetic assimilation rate, proline uptake, and secondary metabolites production	Jangid and Dwivedi (2017)
Apple	Enhanced drought stress tolerance by regulating the leaf photosynthesis and antioxidant enzymes activity	Kumari and Thakur (2019)
Cucumber	Increased drought stress tolerance, improved chlorophyll content, and maintained redox homeostasis	Wei et al. (2015)
Pepper	Decreased excessive ROS accumulation, balanced osmotic adjustment, and increased enzymes activity	Khamsuk et al. (2018)

Drought treatment considerably decreased pepper plant growth status and caused oxidative damage. In contrast, 24-epibrassinolide application significantly mitigated the oxidative damage caused by drought stress. In addition, the 24-epibrassinolide treatment elevated endogenous nitric oxide levels and regulated antioxidant defense mechanisms in pepper plants (Kaya et al., 2019). The total soluble solid content, proline uptake, pigments concentration, and leaf gas exchange elements were considerably improved, while the excessive production of H_2_O_2_, EL, and MDA content were decreased after brassinosteroids treatment (Khamsuk et al., 2018; Siddiqi and Husen, 2021). Foliar application of brassinosteroids effectively increased pigments concentration, chlorophyll content, growth attributes, seedling growth, chlorophyll fluorescence elements, and proline concentration, whereas there was decreased MDA and H_2_O_2_ concentration in *Leymus chinensis* under drought treatment (Lv et al., 2020). The exogenous use of brassinosteroids alleviated the adverse impacts of drought and enhanced drought tolerance by stimulating the antioxidant enzymes activity, fruit production, and pigments concentration in tomato leaf under drought stress (Jangid and Dwivedi, 2017). Kumari et al. (2020) described that brassinosteroids application significantly sustained essential growth and physiological–biochemical activities under drought stress environment. In addition, foliar application of brassinosteroid prior to the onset of stress may mitigate the adverse effects of drought stress on apple plants. Brassinolide enhances the physiological and biochemical characteristics by boosting the antioxidant system and photosynthetic efficiency in *Brassica juncea*. The increased synthesis of proline, enhancement of the antioxidant system, and decreased stress markers provide resilience to plants in coping with stress environment (Naveen et al., 2021). Under drought stress environment, foliar application of brassinosteroids on pepper seedlings effectively preserves vegetative characteristics, mitigates the adverse effects of stress, and diminishes stress indicators (Khosravi and Haghighi, 2021). In a recent study, Zhou et al. (2024) suggested that exogenous brassinosteroids regulates drought tolerance and elucidates the unique roles of *CqBIN2* in the regulation of drought resistance in plants. The author described that ethylene was implicated in brassinosteroid-induced alternative oxidase enzymes activity, which is crucial for tolerance to abiotic stressors in cucumber seedlings (Wei et al., 2015).

**Figure 6:**
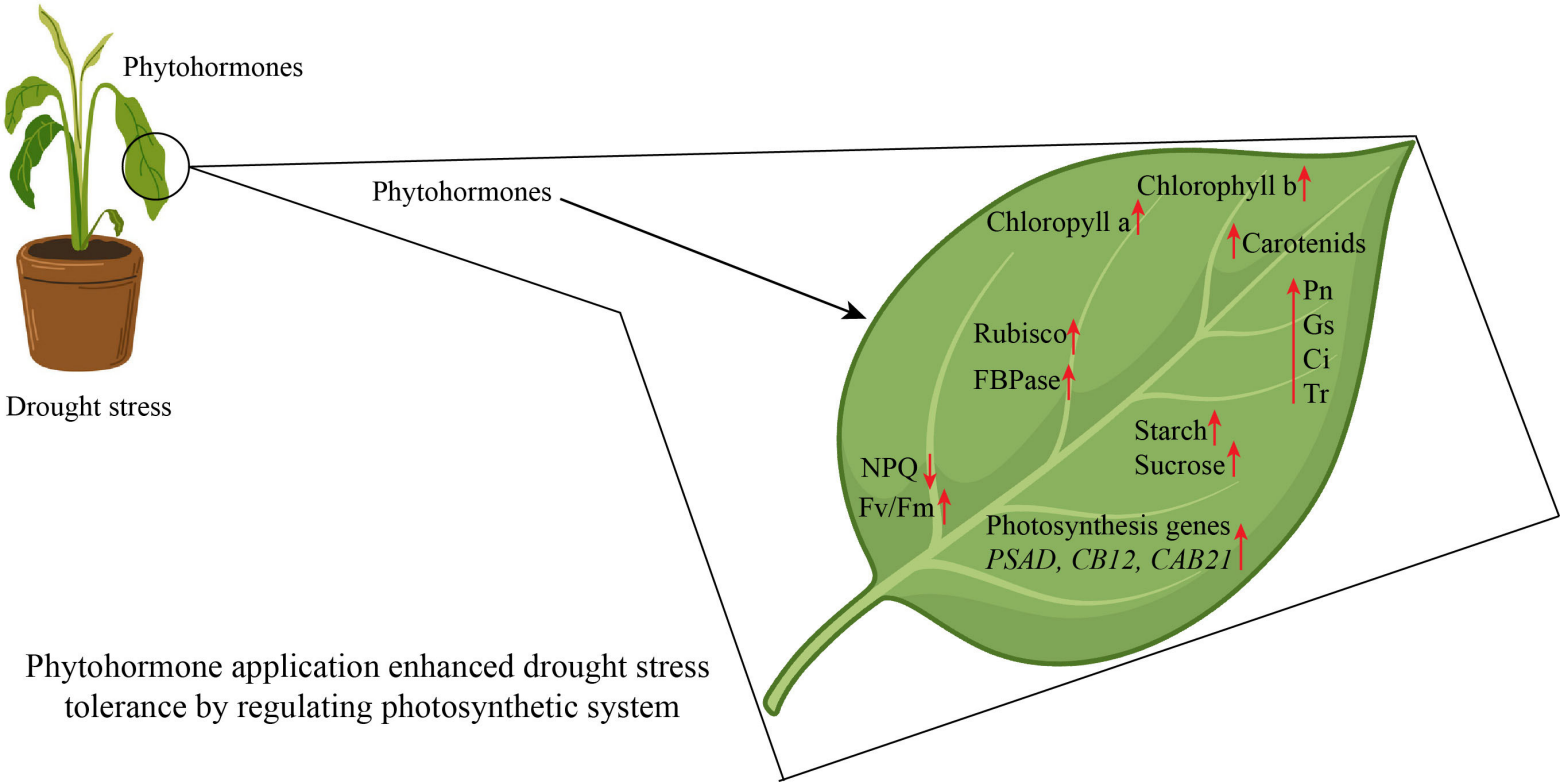
Phytohormone application protected photosynthetic apparatus in horticultural plants. NPQ, non-photochemical quenching; Pn, Net.

Brassinosteroids application potential enhanced the secondary metabolites accumulation in pepper leaf (Samancıoğlu et al., 2014), protected photosynthetic capacity in apple leaf (Kumari and Thakur, 2019), regulated nitrogen and antioxidant defense mechanism in potato (Guo et al., 2024), enhanced carbohydrates and total soluble sugar concentration in radish (Balaraju et al., 2015), and decreased excessive ROS accumulation in cucumber (Xia et al., 2009). The author clearly indicates that local brassinosteroids application may stimulate the sustained generation of H_2_O_2_, and the self-propagating characteristic of the ROS signal subsequently facilitates EBR-induced systemic tolerance in cucumber (Xia et al., 2011). Zheng et al. (2022) described that leaf water content, chlorophyll content, pigments concentration declined, while brassinosteroids application signifcantly increased the leaf water status and photosynthetic assimilation rate and reduced oxidative damage in potato plants under drought stress. Exogenous 24-EBL application significantly increased the growth and petioles elongation in carrot (Que et al., 2017). The radish seedling growth and seed germination were increased with Brassinosteroids supplementation under water stress environment. In addition, the antioxidant enzymes activity, chlorophyll content, and proline uptake were increased, while MDA accumulation, H_2_O_2_ production, and EL level were considerably decreased in radish seedling under water stress conditions (Mahesh et al., 2013). Brassinosteroids are essential phytohormones that modulate signals to improve resilience to stress in plants. The results suggest that brassinosteroids are potentially beneficial, eco-friendly, naturally occurring compounds that may be extensively used to mitigate the impacts of abiotic stress (**Figure 6**).

### Strigolactones

Strigolactones are derivatives of carotenoid that respond to different environmental stimuli by acting as both endogenous and external signaling molecules (Pandey et al., 2016). The strigolactones have a positive influence on horticultural plants (Siddiqi and Husen, 2017). Strigolactones regulate different functions in horticultural plants such as seed germination, flowering, seedling growth, cell elongation, photosynthesis, hormonal production, fruit ripening, redox balanced, antioxidant enzymes activity, secondary metabolites production, and leaf water potential in horticultural plants (**Table 6**) (Kaniganti et al., 2022; Sharma et al., 2024). Additionally, strigolactones cause the vascular cambium to become more active meristematically, which promotes secondary growth (Naseer et al., 2024). Strigolactones are associated with chlorophyll synthesis (Jiang et al., 2024). Under low light, plants produce more auxin, which enhances the synthesis of strigolactones. Strigolactones enhance root hair elongation but prevent the formation of interfascicular cambium in buds. Numerous functions of strigolactones has been described such as root system architecture, root morphology, plant defense mechanisms, and nutrient absorption (Soliman et al., 2022). Strigolactones serve as multifaceted signaling molecules, regulating numerous plant metabolic functions, including tolerance to drought stress (Makhzoum et al., 2017). Strigolactones were first recognized for their function in stimulating germination in root-parasitic plants (Khalid et al., 2024). Strigolactones provide the potential to improve our capacity to safeguard plants against the effects of drought stress.

**Table 6:** Strigolactones enhanced drought stress tolerance in horticultural crops.

Crops	Findings/plant responses	References
Tomato	Increased pigments concentration and enhanced drought tolerance and antioxidant oxidant enzymes activity	Baltacıer et al., 2023
Grapevine	Enhanced drought stress tolerance, maintained osmotic adjustment, and reduced oxidative damage in leaf	Min et al., 2019
Lettuce	Enhanced drought stress tolerance, promoted mineral homeostasis, maintained osmotic adjustment, and protected chlorophyll content	Ruiz-Lozano et al., 2016
Pepper	Maintained redox homeostasis, increased photosynthesis, lowered H_2_O_2_, and MDA accumulation in pepper leaf	Shu et al., 2023
Apple	Enhanced antioxidant enzymes activity, lowered oxidative damage, and increased chlorophyll content	Xu et al., 2023
Cucumber	Increased drought stress tolerance, osmotic adjustment, maintained redox homeostasis, and lower EL level in cucumber leaf	Zhou et al., 2022

Foliar application of strigolactones signifcantly protected the photosynthetic apparatus, regulated antioxidant defense mechanism, maintained redox homeostasis, and enhanced drought stress tolerance in pepper (Shu et al., 2023). Strigolactones (GR24) pretreatment mitigates the detrimental effects of drought stress on grapevine seedlings. Under drought stress, strigolactones (GR24) might more effectively promote stomatal closure. Strigolactones (GR24) may regulate chlorophyll constituents and mitigate the reduction of photosynthesis caused by dryness. The interaction of strigolactones with other hormones, particularly abscisic acid, may represent a significant factor in drought response (Min et al., 2019). The foliar application of strigolactones may positively influence the responses of *Brassica rapa* plants under drought environment (Ali et al., 2023). Furthermore, strigolactones treatment enhances the photosynthetic efficiency of *Pennisetum purpureum* leaves under drought conditions and elevates the antioxidant capacity of the leaves, thereby mitigating the detrimental effects of drought, fostering the growth of *Pennisetum purpureum*, and significantly enhancing its drought resistance (Li Y. et al., 2022). Strigolactones (GR24) substantially alleviates the drought-induced damage caused in apple. In addition, strigolactones (GR24) mitigate the drought-induced reduction in photosynthesis via modulating pigment molecules concentration and stomatal aperture. Strigolactones (GR24) mitigate oxidative damage by increasing antioxidant defense system. Strigolactones (GR24) improve drought resistance in apple via activating the expression of Ca^2+^ signaling-associated genes (Xu et al., 2023).

Strigolactones application positively regulates the growth of cucumber (Zhou et al., 2022), modulates the photosystem II efficiency in tomato and lettuce (Ruiz-Lozano et al., 2016), upregulates antioxidant enzymes activity in tomato (Baltacıer et al., 2023), and maintains redox homeostasis and reduces oxidative damage in pepper (Shu et al., 2024). Omoarelojie et al. (2020) reported that strigolactone-pretreated lupine seeds exhibited enhanced germination and seedling development, along with elevated proline levels and reduced MDA concentration. Foliar application of strigolactones markedly enhanced stomatal sensitivity in tomato plants under drought stress environment (Visentin et al., 2016). Furthermore, the supplementation of strigolactones enhanced the activity of the glyoxalase system and antioxidant enzymes in lupine seedlings. Furthermore, strigolactones modulate many hormone-responsive pathways, enabling plants to overcome environmental stressors and mitigate adverse effects on horticultural crop productivity (Bhoi et al., 2021). In response to various environmental stresses, strigolactones appear to be slightly important in the stress physiology of horticultural plants.

### Gamma-aminobutyric acid

Gamma-aminobutyric acid (GABA) is a newly discovered plant growth regulator (Hasan et al., 2021). GABA regulated secondary metabolites accumulation, maintained redox homeostasis, upregulated antioxidant enzymes activity, balanced mineral accumulation, protected photosynthetic apparatus, and enhanced seedling growth in horticultural plants (Sita and Kumar, 2020). GABA protects plants from drought stress by boosting secondary metabolites production and leaf turgor while lowering oxidative damage via regulation of antioxidant defense system (Kinnersley and Turano, 2000). The application of GABA may enhance the growth and production of pepper under drought stress condition. Furthermore, foliar application of GABA enhanced secondary metabolites accumulation and the activity of antioxidant enzymes associated with pepper plant defense mechanisms (Iqbal et al., 2023). The supplementation of GABA under water stress in snap bean plants enhanced field performance, shown by upregulation of antioxidant enzymes activity, and maintained cellular membrane integrity level, higher pod production, and quality traits. In conclusion, exogenous GABA serves as an efficient priming agent to mitigate drought-induced oxidative damage in snap bean plants under drought stress condition (Abd El-Gawad et al., 2021). GABA application enhanced seed germination, osmolytes production, and antioxidant enzymes activity in white clover under drought stress (Zhou et al., 2021). The foliar treatment of GABA significantly enhanced the drought stress tolerance of cucumber seedlings by elevating antioxidant enzymes activity, free proline concentrations, protected photosynthetic capacity, and leaf relative water content. In addition, GABA treatment may serve as an effective approach to mitigate the detrimental impacts of drought stress on cucumber cultivation (Ghahremani et al., 2023). GABA application enhanced pigments concentration, soluble sugar content, protein concentration, and secondary metabolites production in pea leaves under drought stress environment (Al-Quraan et al., 2021). Cheng et al. (2023) reported that foliar application of GABA improved photosynthetic assimilation rate, increased leaf water content, and reduced EL level in apple leaves (**Table 7**).

**Table 7:** GABA enhanced drought stress tolerance in horticultural crops.

Crops	Findings/plant responses	References
Tomato	Regulated secondary metabolites production and enhanced drought stress tolerance	Tilahun et al. (2021)
Black pepper	Enhanced antioxidant enzymes system and upregulated proline and soluble sugar content	Vijayakumari and Puthur (2016)
Apple	Decreased electrolyte leakage, ROS production, and increased leaf photosynthetic capacity	Cheng et al. (2023)
Cucumber	Increase seed germination rate, improved antioxidant system, and regulated drought stress tolerance	Zahra et al. (2021)
Pear fruit	Enhanced GAD enzymes activity and regulated polyamine metabolism	Liu et al. (2022)
Muskmelon	Increased nitrogen metabolism and enhanced seedling growth and stress tolerance mechanism in plants	Zhen et al. (2018)

### Crosstalk among phytohormones

**Figure 7:**
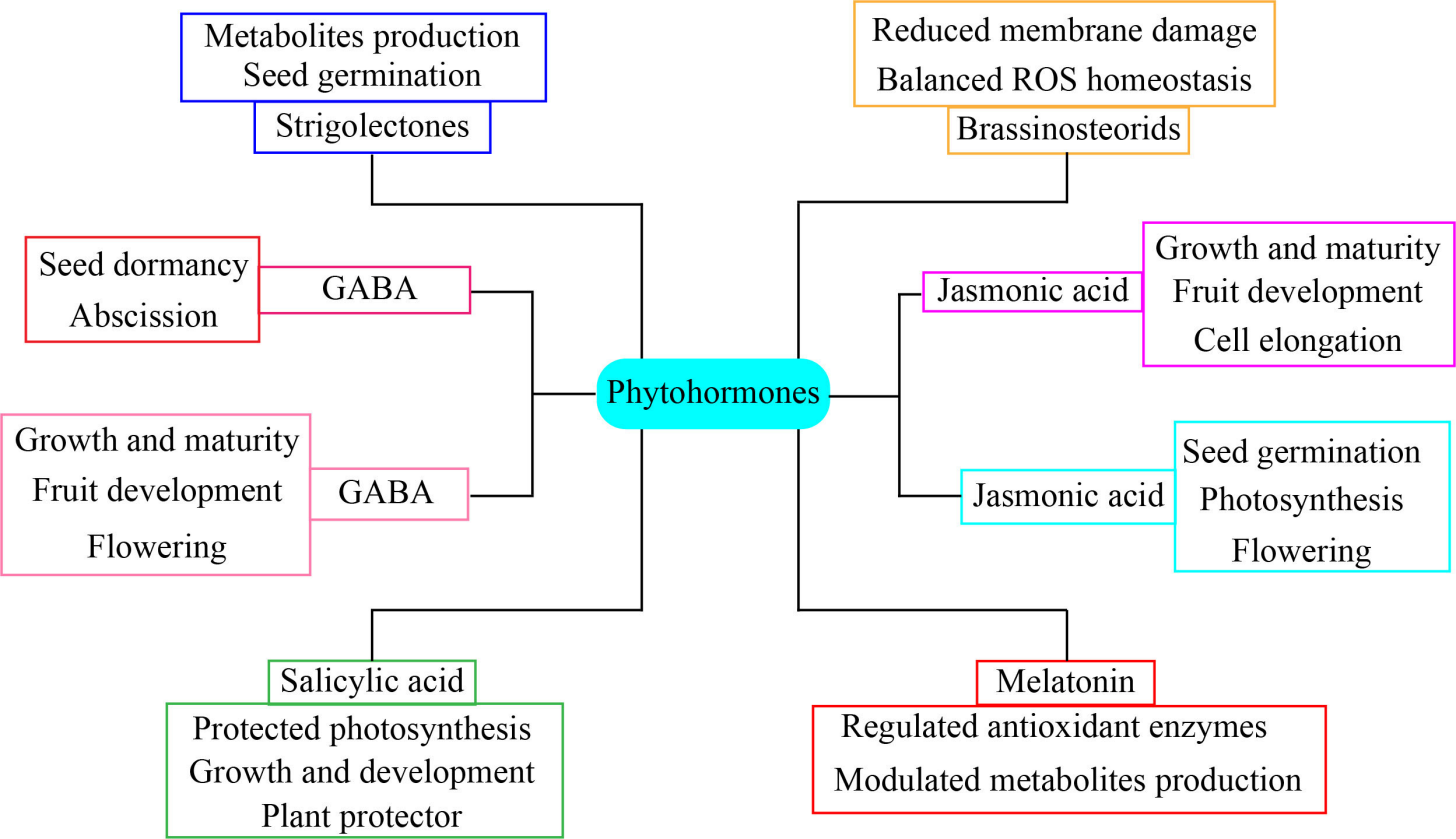
Phytohormone crosstalk application enhanced drought stress tolerance in horticultural plants.

The phytohormones participate in the interaction with other growth regulators, resulting in notable alterations in the phenology of horticultural plants (**Figure 7**). Abscisic acid has a crucial function in regulating situations of drought stress (Kleman and Matusova, 2023). Stomatal closure is a significant morphological characteristic that is rigorously maintained and mostly influenced by drought stress (Ciura and Kruk, 2018). Under drought stress, abscisic acid application along with jasmonic acid and nitric oxide promote stomatal closure (Altaf et al., 2023). The interaction between the salicylic acid pathway and the abscisic acid, jasmonic acid, and ethylene pathways is essential for the regulation of plant growth and stress responses (Jogawat et al., 2021). Furthermore, the combination of salicylic acid and melatonin significantly enhances the drought tolerance of tomato plants. The combination hormone treatment considerably increased antioxidant enzymes activity, promoted hormone production, and maintained methylglyoxal enzymes pool, which increased the tolerance of tomato plants to drought stress (Kaya et al., 2023). Drought stress has the greatest detrimental impact on lettuce productivity. The combined salicylic acid and melatonin improved lettuce resilience to drought stress. The molecular mechanisms and biochemical interactions of melatonin and melatonin-mediated phytohormonal crosstalk in horticultural plants play a multifaceted role in enhancing drought stress tolerance (Dzinyela et al., 2024). Phytohormones are essential molecules that facilitate drought tolerance, thus offering new opportunities for the preservation of sustainable crop yields for addressing global food demand in the face of changing the environment (Tiwari et al., 2017). Phytohormones coordinate essential developmental signals and transmit environmental information via synergistic or antagonistic interactions known as signaling crosstalk (Seif El-Yazal et al., 2015). Gibberellic acid signaling and its interaction with other hormonal pathways elucidate the multifaceted function of DELLA proteins in conjunction with components of many hormonals signaling pathways (Niharika et al., 2021). Auxin, gibberellins, and cytokinins are pivotal in controlling development under stress situations, whereas abscisic acid and ethylene inhibit growth by modifying the actions of GA, auxin, and CK under adverse conditions (Ullah et al., 2018). The interplay between brassinosteroids (BRs) and gibberellins (GAs) is well established. Mutant BR signaling in *Arabidopsis thaliana* showed many changes in the expression of GA biosynthetic genes, which might be because bioactive GA synthesis was disrupted (Bano et al., 2023). Auxin, an additional hormone, stimulates hypocotyl development. The auxin signaling pathway entails the modulation of transcription factor auxin response factors via the degradation of AUX/IAA family members (Emenecker and Strader, 2020). The connectivity between GA and ABA facilitates the balance between seed germination and dormancy, which is crucial for stress tolerance. These hormones have an antagonistic connection, resulting in elevated GA and diminished ABA levels under favorable circumstances and reduced GA and increased ABA levels under adverse environmental conditions in seeds (Altaf et al., 2023). The combined application of salicylic acid and melatonin more efficiently mitigates stress in lettuce. In addition, salicylic acid application along with melatonin enhanced the nutritional status vitamin C and antioxidant potential and reduced nitrate concentration in lettuce (Kiremit et al., 2024). Melatonin application crosstalk with other phytohormone significantly enhanced stomatal regulation and photosynthetic capacity in horticultural plants (Sun et al., 2023). The advancement of mechanistic methodologies is essential to mitigate the detrimental impacts of drought on horticultural crops.

## Conclusion and future prospects

Plants require sophisticated signaling pathways to regulate and develop in a variety of environmental conditions. Horticultural crops have increased vulnerability to variable environmental conditions. Phytohormones are compounds that affect the vegetative and reproductive development of plants while alleviating different abiotic stressors. These dynamic molecules substantially modify the metabolic fluxes inside the plant cell under stress circumstances to promote a more resistant phenotype. Phytohormones are essential stress mitigator throughout all phases of crop growth. Information accumulates about the advantageous impacts of phytohormones like salicylic acid, melatonin, brassinosteroids, jasmonate, and strigolactones in horticultural crops. Substantial data indicate that phytohormone responses vary across different phases of organ development, perhaps owing to distinct cellular and tissue contexts. A complex network of phytohormones affects root shape, emphasizing the need of understanding transcriptional and post-transcriptional processes and their genes. These investigations will enhance understanding of how roots detect internal and external signals and convert them into cellular responses while also allowing breeders to develop predictive models to identify crucial regulators and integrators of root system architecture under different environmental circumstances. Future investigations into phytohormone interactions with other signaling molecules for drought resilience in horticulture crops should concentrate on many critical domains as follows:

- Exploring the precise molecular processes by which phytohormones interact with other signaling molecules to help various horticulture crops tolerate drought.
- Finding and analyzing novel phytohormones and signaling molecules linked to drought tolerance and comprehending how they interact with another pathway.
- Establishing new instruments and technologies, as genome editing and sophisticated imaging methods, to investigate complicated signaling pathways connected to drought tolerance.
- Investigating how temperature and light affect the way that phytohormones interact with other signaling molecules to help plants withstand drought.

Future study in these domains might substantially enhance our comprehension of the molecular processes underlying drought tolerance in horticulture crops and facilitate the identification of novel ways for boosting agricultural yields and securing global food security amid climate change.
